# A large close relative of *C. elegans* is slow-developing but not long-lived

**DOI:** 10.1186/s12862-019-1388-1

**Published:** 2019-03-11

**Authors:** Gavin C. Woodruff, Erik Johnson, Patrick C. Phillips

**Affiliations:** Department of Biology, Institute of Ecology and Evolution, University of Oregon, Eugene, USA

**Keywords:** Body size, Life history, *C. elegans*, Lifespan, Heterochrony

## Abstract

**Background:**

Variation in body size is thought to be a major driver of a wide variety of ecological and evolutionary patterns, including changes in development, reproduction, and longevity. Additionally, drastic changes in natural context often have profound effects on multiple fitness-related traits. *Caenorhabditis inopinata* is a recently-discovered fig-associated nematode that is unusually large relative to other members of the genus, including the closely related model system *C. elegans*. Here we test whether the dramatic increase in body size and shift in ecological context has led to correlated changes in key life history and developmental parameters within this species.

**Results:**

Using four developmental milestones, *C. inopinata* was found to have a slower rate of development than *C. elegans* across a range of temperatures. Despite this, *C. inopinata* did not reveal any differences in adult lifespan from *C. elegans* after accounting for differences in developmental timing and reproductive mode. *C. inopinata* fecundity was generally lower than that of *C. elegans*, but fitness improved under continuous-mating, consistent with sperm-limitation under gonochoristic (male/female) reproduction. *C. inopinata* also revealed greater fecundity and viability at higher temperatures.

**Conclusion:**

Consistent with observations in other ectotherms, slower growth in *C. inopinata* indicates a potential trade-off between body size and developmental timing, whereas its unchanged lifespan suggests that longevity is largely uncoupled from its increase in body size. Additionally, temperature-dependent patterns of fitness in *C. inopinata* are consistent with its geographic origins in subtropical Okinawa. Overall, these results underscore the extent to which changes in ecological context and body size can shape life history traits.

**Electronic supplementary material:**

The online version of this article (10.1186/s12862-019-1388-1) contains supplementary material, which is available to authorized users.

## Background

Trade-offs dominate life history evolution. Organisms have access to limited energy resources, and these must be allocated in a balance between self-maintenance and reproductive output. In keeping with the expectation that different distributions of life history traits (such as age of maturity, reproductive duration, and age-specific fecundity, among others) should be sensitive to different distributions of selective pressures on those traits, a huge diversity of patterns among life history traits has emerged across the broad scope of animal diversity [1–5]. As a consequence, many organisms exhibit well-documented correlations among traits such as fecundity and survival [6–8], fecundity and developmental rate [1, 9–11], and reproductive quantity and quality [12, 13].

Body size is a particularly potent component of life history syndromes. Body size is usually correlated with a multitude of fitness-related traits including developmental rate, offspring number, offspring size, gamete size, and lifespan [14–17]. Body size is also known to covary with physiological traits, such as metabolic rate, thought to underlie trade-offs among life history traits [15, 17]. These factors in turn generate allometric relationships that appear to explain scale-based trends for a wide variety of traits across many orders of magnitude [15]. Indeed, body size appears to be a central component of broad macroevolutionary trends among lineages over geological timescales [18]. But which is cause and which is effect? To what extent does change in body size due to selection on body size per se lead to collected changes in such a wide array of life history traits and to what extent does body size change because of selection acting directly on these traits?

Life history theory suggests that selection for increased body size can be balanced against the benefits of faster reproduction and the costs of lower offspring viability and lower initial fecundity [1], weighed against a backdrop of differential allocation of physiological and metabolic resources to each of these processes and to growth itself [17, 19]. At the same time, selection on body size itself must be mediated via environmental factors such as resource availability and/or predation [20]. Although these various causes are not mutually exclusive and likely overlap, the proximate and ultimate causes of body size change—particularly the relationship between these two—remain largely unresolved.

However, body size is not the only factor known to influence life history traits—environmental and ecological change is also expected to promote life history evolution. Indeed, whenever a change in environment impacts the optimal survival and fecundity of different age classes, then life histories will evolve in response [21]. Changes in both abiotic (temperature, salinity, humidity, etc.) and biotic (predation pressure, resource abundance, pollinator density, etc.) environments can impact life history strategies: for instance, differential predation on larval stages in guppies due to spatial differences in predator abundance promoted changes in reproductive effort across populations [21, 22]. Furthermore, the extent of regularity in temporal environments (such as season length) is thought to underlie bet-hedging strategies (such as the germination time decision), and life histories are expected to evolve in response to changes in the timing of environmental cycles as well [21]. Thus, environmental and ecological context plays a critical role in the evolution of life histories. How do changes in ecological context interact with the constraints imposed by body size to promote the evolution of life history traits?

The nematode *Caenorhabditis elegans* has for decades been an important model for genetics, development, and biology in general [23]. However, the degree and extent of trade-offs between body size and other life history traits in systems like *C. elegans* remain largely unknown and/or have generated somewhat ambiguous or contradictory results [24–32]. Further, because nearly all known members of this genus share a common natural ecological niche of rotting plant material [33], it has not been possible to use a comparative approach to investigate how change in ecological circumstances might drive changes in the relationship between body size and life history [19]. Here, we address this question by taking advantage of a highly phenotypically and ecologically divergent close relative of *C. elegans*: the recently discovered fig-associated nematode *C. inopinata*.

*C. inopinata* (formerly known as *C.* sp. 34) is remarkable in that it displays tremendous ecological and phenotypic differences compared to its close relatives [34, 35]. Compared to other *Caenorhabditis*, *C. inopinata* is huge: it can grow to be nearly twice as long as other members in the genus [34, 35]. *C. inopinata* also develops nearly half as quickly, has sperm three times the size, and embryos 20% longer than *C. elegans* [35]. Furthermore, in contrast to the rotting-plant material ecological niche of *C. elegans* and other *Caenorhabditis* species [36], it thrives in the fresh, intact Okinawan figs of *Ficus septica* [34, 35, 37]. *C. inopinata* thus appears to have experienced a radically different selective environment that has led to its highly divergent suite of life history traits. And, as *C. inopinata* is much larger in size and develops much more slowly than its close relatives, it can therefore be used as a natural system to test the predictions of life history theory using a comparative approach. Here, we do just this by describing the developmental timing, lifespan, fecundity, and viability of *C. inopinata* and *C. elegans* at multiple temperatures.

## Results

### *C. inopinata* develops more slowly yet does not differ from *C. elegans* in lifespan and reproductive duration

**Fig. 1:**
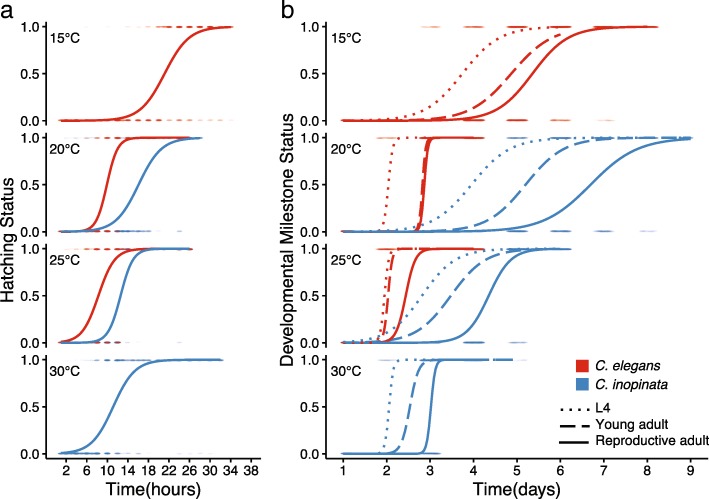
*C. inopinata* develops more slowly than *C. elegans*. The y-axis represents the status of having attained a given developmental milestone; 0 = has not reached milestone, 1 = has reached milestone. Here, the actual data representing animals at (or not at) developmental milestones are plotted as clouds of points at these values over time. The curves are logistic models of growth that were fit to these data (see Additional files 5 and 6 for data and Additional file 4 for software to generate these models). **a**) Hatching; **b**) L4, young adulthood, and the onset of reproduction. *C. elegans (fog-2)* was used for the embryogenesis milestone to account for the delay caused by obligate outcrossing in *C. inopinata*. *C. elegans* N2 is inviable at 30°C, and *C. inopinata* milestones were not measured at 15°C due to its low fitness at this temperature. N worms=385, *C. elegans* hatching 15°C; N=417, *C. inopinata* hatching 20°C; N=320, *C. elegans* hatching 20°C; N=383, *C. inopinata* hatching 25°C; N=319, *C. elegans* hatching 25°C; N=437, *C. inopinata* hatching 30°C; N=225, *C. elegans* L4 15°C; N=186, *C. inopinata* L4 20°C; N=270, *C. elegans* L4 20°C; N=209, *C. inopinata* L4 25°C; N=263, *C. elegans* L4 25°C; N=232, *C. inopinata* L4 30°C; N=225, *C. elegans* young adult 15°C; N=186, *C. inopinata* young adult 20°C; N=270, *C. elegans* young adult 20°C; N=209, *C. inopinata* young adult 25°C; N=263, *C. elegans* young adult 25°C; N=232, *C. inopinata* young adult 30°C; N=714, *C. elegans* reproductive adult 15°C; N=380, *C. inopinata* reproductive adult 20°C; N=677, *C. elegans* reproductive adult 20°C; N=784, *C. inopinata* reproductive adult 25°C; N=960, *C. elegans* reproductive adult 25°C; N=527, *C. inopinata* reproductive adult 30°C. GLM LRT chi-square p<0.0001 for every *C. elegans* and *C. inopinata* comparison.

Initial measures of developmental rate revealed that *C. inopinata* develops at about half the rate as *C. elegans* [35]. To provide a more complete picture of the timing of development in this species, the occurrence of four different developmental milestones (time of hatching, onset of the L4 stage, onset of adulthood, and the onset of reproduction) was ascertained at four different temperatures (15°C, 20°C, 25°C, and 30°C) among synchronized populations of *C. elegans* and *C. inopinata*. Unsurprisingly, all species grew faster as the temperature increased (Fig. 1; Additional file 1: Table S1). Yet in conditions where both species grew reliably, *C. inopinata* was slower to reach all developmental milestones than *C. elegans* (Fig. 1; Additional file 1: Table S1). Indeed, at the typical rearing temperature of *C. elegans* (20°C), the median time of reproductive onset was 2.7 days in *C. elegans*, whereas it was 6.7 days in *C. inopinata* (Generalized linear model likelihood ratio test (GLM LRT) chi-square=4861.4, df=2, p<0.0001). To reach a developmental rate that approaches that of *C. elegans* at 20°C, *C. inopinata* must be reared at a temperature that is ten degrees higher (Fig. 1b; Additional file 1: Table S1) where it exhibits reduced fecundity (Fig. 4a) and where *C. elegans* N2 is inviable (Fig. 5). Overall, then, *C. inopinata* has slower relative growth regardless of temperature.

**Fig. 2:**
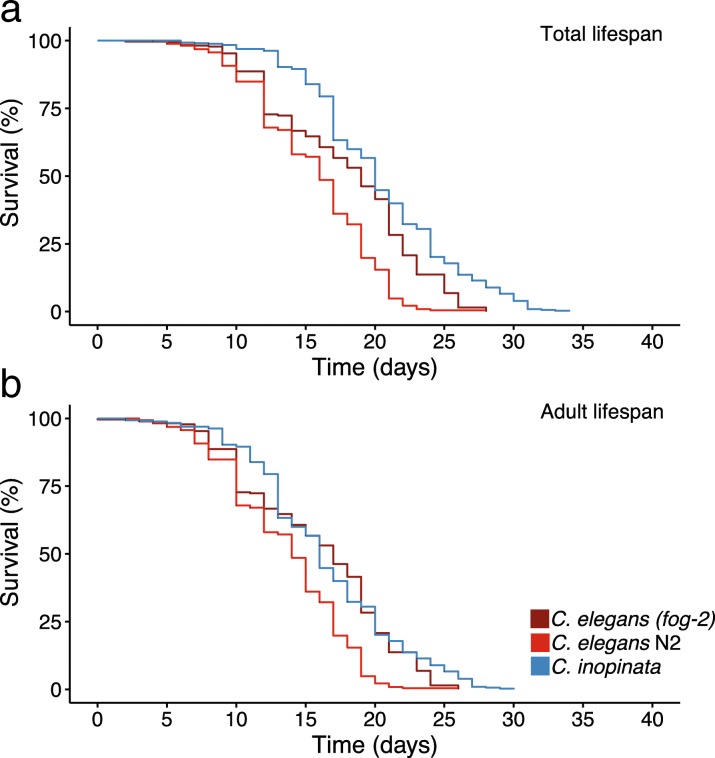
*C. inopinata* is not longer-lived than *C. elegans* at 25°C after taking reproductive mode and developmental timing into account. (**a**) Total lifespan models. Here, Day = 0 represents the day embryos were laid. (**b**) Adult lifespan models. Here, Day = 0 is the approximate first day of adulthood, taken as the total lifespan minus two (*C. elegans*) or four (*C. inopinata*) days. Wild-type *C. elegans* N2 exhibits both shorter total and adult median lifespan than *C. inopinata*. Conversely, *C. inopinata* females have a marginally higher median total lifespan than non-selfing *C. elegans (fog-2)* mutant females, and no difference in *C. inopinata* and *C. elegans (fog-2)* adult lifespan was detected (Cox proportional hazards linear model comparison, Z-value=0.74, p=0.73). N worms=263 (*C. elegans* N2), N=281 (*C. elegans (fog-2)*), N=444 (*C. inopinata*).

As slow developing, large animals tend to be longer-lived [1], we were curious if *C. inopinata* also exhibits prolonged longevity. To address this, we applied previously established methods of lifespan measurement in nematodes [38] to *C. inopinata*. As a point of comparison, we also measured *C. elegans* N2 and *C. elegans* (*fog-2*; for Feminization Of Germline) lifespans. As lifespan often trades-off with reproductive output [39, 40], we used virgin *C. elegans (fog-2)* pseudo-females (which do not generate self-sperm and are self-sterile as a consequence [41]) to control for differences in reproductive mode. *C. inopinata* females were longer-lived than wild-type *C. elegans* hermaphrodites at 25°C, with a median total lifespan that was four days higher (20 and 16, respectively; Cox proportional hazards linear model comparison, Z-value=4.99, p<0.0001 Fig. 2a; Additional file 1: Figure S1). However, *C. inopinata* females were only marginally longer lived than *C. elegans (fog-2)* pseudo-females (19 days, Cox proportional hazards linear model comparison, Z-value=2.29, p=0.053). Furthermore, no differences in adult lifespan (which takes into account the differences in developmental timing between *C. elegans* and *C. inopinata*) were detected between *C. inopinata* females (median adult lifespan of 16 days) and *C. elegans (fog-2)* pseudo-females (median adult lifespan of 17 days; Cox proportional hazards linear model comparison, Z-value=0.74, p=0.73; Fig. 2b; Additional file 1: Figure S2). Thus, despite its large size and slow development, *C. inopinata* adults are not longer-lived than *C. elegans* after accounting for differences in reproductive mode and developmental timing.

**Fig. 3:**
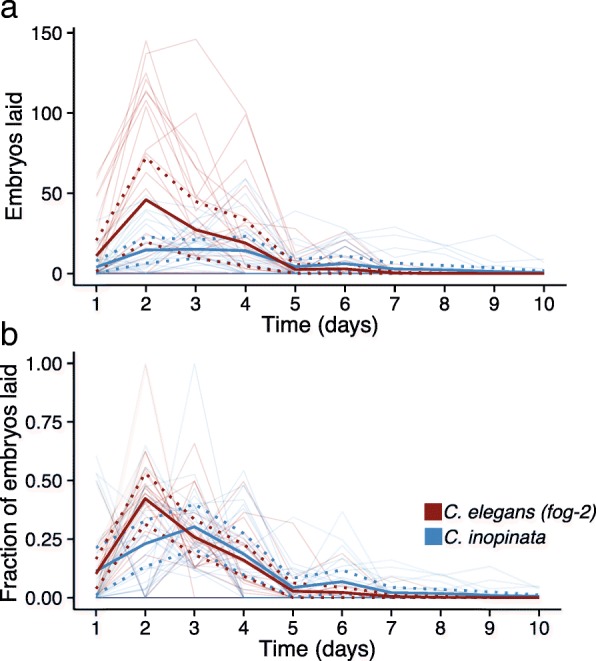
*C. inopinata* has a reproductive duration comparable to *C. elegans*. (**a**) Number of embryos laid per day. (**b**) Fraction of lifetime brood laid per day. Bold lines represent averages, and dotted bold lines represent ±1 SDM. Thin lines represent individual worms. The *C. elegans (fog-2)* and *C. inopinata* day two and three brood fractions are not statistically different (Wilcoxon rank sum test W=389 p=0.36 and W=553 p=0.13, respectively). N parental females=30 for both species. All observations taken at 25°C.

The duration of reproduction is also expected to trade-off with growth rate and body size [1, 2], with large, slow-developing animals tending to have longer reproductive periods [9–11]. To see if this also holds for *C. inopinata*, daily measures of fecundity were made with individual *C. elegans* (*fog-2*) pseudo-females and *C. inopinata* females under conditions of continuous mating throughout their lifetimes (Fig. 3). Although one individual *C. inopinata* female had a reproductive duration of twelve days, for the most part, both species lay almost all of their embryos in the first four days of adulthood (Fig. 3b). Indeed, under continuous mating conditions at 25°C, no differences in brood fraction per day could be detected between *C. inopinata* and *C. elegans* with the exception of day eight of adulthood (Wilcoxon rank sum test, W=528, p=0.041). Thus, like lifespan, duration of reproduction is not extended in *C. inopinata*.

### *C. inopinata* is sperm-limited and reveals higher fitness at higher temperatures

**Fig. 4:**
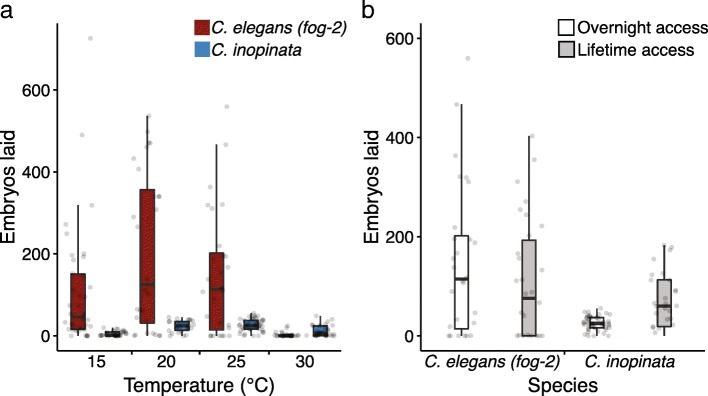
*C. inopinata* is sperm-limited. (**a**) Number of embryos laid in single overnight mating conditions at various temperatures. (**b**) Number of embryos laid in continuous mating or single overnight mating conditions at 25°C. The “one overnight mating” data in panel (**b**) is the same from those at 25°C in panel (**a**). *C. inopinata* has smaller broods than *C. elegans (fog-2)* in every condition except 30°C (Wilcoxon rank sum test p<0.0001 for 15 and 20°C; W=349, p=0.004 for 25°C; W=575, p=0.002 for 30°C). However, there is no detectable difference in *C. elegans (fog-2)* and *C. inopinata* brood sizes under continuous mating conditions (Wilcoxon rank sum test, W=484, p=0.62). N parental females=38, C. elegans overnight access 15°C; N=28, *C. inopinata* overnight access 15°C; N=28, *C. elegans* overnight access 20°C; N=26, *C. inopinata* overnight access 20°C; N=28, *C. elegans* overnight access 25°C; N=42, *C. inopinata* overnight access 25°C; N=28, *C. elegans* overnight access 30°C; N=28, C. inopinata overnight access 30°C; N=30, *C. elegans* lifetime access 25°C; N=30, *C. inopinata* lifetime access 25°C.

Brood size also tends to covary with both body size and developmental rate [1, 2], and so fecundity was measured at four different temperatures in *C. inopinata* and *C. elegans (fog-2)* to address if similar patterns hold in this group (Fig. 4). In conditions in which females were mated with males for just one night, *C. inopinata* generally displayed far smaller brood sizes than *C. elegans (fog-2)*, with the exception that *C. elegans (fog-2)* is infertile at 30°C (Fig. 4a). However, as the male/female species *C. remanei* is known to generate more progeny when constantly exposed to males [42, 43], we suspected that *C. inopinata* might also be sperm-limited. Indeed, under continuous mating conditions, there is no detectable difference in brood size between *C. inopinata* and *C. elegans (fog-2)* (median brood size of 58 and 76, respectively; Wilcoxon rank sum test, W=484 p=0.62; Fig. 4b). However, male mating performance tends to degrade in selfing species [44], so we also compared the fraction of successful crosses between *C. elegans* and *C. inopinata* (Additional file 1: Figure S3). In continuous mating conditions, the fraction of failed crosses was higher in *C. elegans* (0.33, N=30 crosses) than in *C. inopinata* (0.17, N=30 crosses), although this difference was not statistically significant (Fisher’s Exact Test odds ratio=2.46, p=0.23). After removing animals that failed to produce progeny, *C. elegans (fog-2)* yielded a median brood size that is over twice as large as that of *C. inopinata* in continuous mating conditions (145 and 65, respectively; Wilcoxon rank sum test, W=359, p=0.013; Additional file 1: Figure S4). Thus *C. inopinata* requires constant access to mates in order to maximize its reproductive output, consistent with its gonochoristic mode of reproduction.

When examining the relationship between developmental rate and fecundity, the intrinsic rate of increase (*r*) is likely a better measure of fitness than total fecundity (*R*_0_) [1, 45]. Under this approach, fitness is a function of age-specific fecundity and viability, and the age of first reproduction can highly influence the population growth rate [1]. So although *C. inopinata* and *C. elegans* have comparable brood sizes under continuous mating conditions, they likely differ in fitness because of their different developmental rates. Indeed, despite their comparable brood sizes, *C. elegans* has a rate of increase (*r*=1.54, 95% confidence interval (CI)=1.26-1.72) that is over twice as high as *C. inopinata* (*r*=0.66, 95% CI=0.54-0.74). This difference in fitness is even greater in mating conditions with just overnight access to males (*C. elegans r*=2.09, 95% CI=1.88-2.24; *C. inopinata r*=0.63, 95% CI=0.55-0.69). Thus continuous access to males is not sufficient to overcome the detriment to fitness due to slow development in *C. inopinata*.

**Fig. 5:**
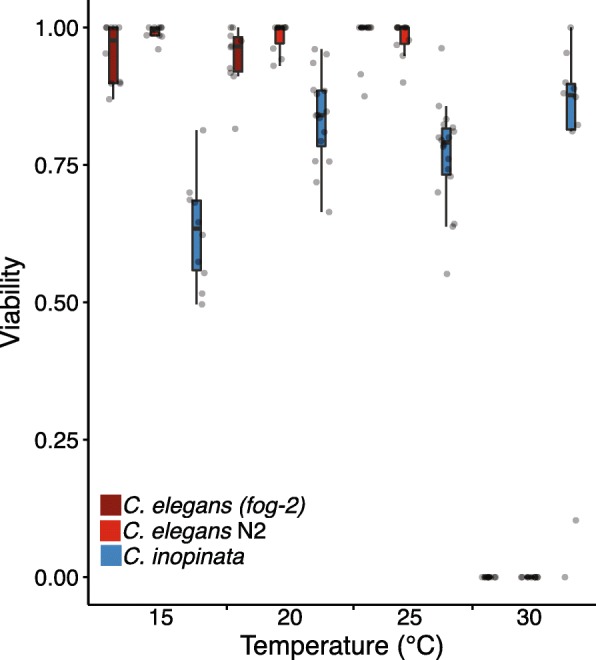
*C. inopinata* has a lower viability than *C. elegans*. Embryo-to-adult viability at four temperatures. *C. elegans* reveals higher viability in all conditions except 30°C regardless of reproductive mode. N plates=10, *C. inopinata* 15°C; N=10, *C. elegans* N2 15°C; N=10, *C. elegans (fog-2)* 15°C; N=16, *C. inopinata* 20°C; N=10, *C. elegans* N2 20°C; N=10, *C. elegans (fog-2)* 20°C; N=18, *C. inopinata* 25°C; N=10, *C. elegans* N2 25°C; N=10, *C. elegans (fog-2)* 25°C; N=10, *C. inopinata* 30°C; N=10, *C. elegans* N2 30°C; N=10, *C. elegans (fog-2)* 30°C; N embryos per plate=5-237.

In keeping with the other life-history measures, *C. elegans* was more viable at lower temperatures and *C. inopinata* more viable at higher temperatures during early development (Fig. 5). Overall, however, *C. inopinata* displayed consistently lower embryo-to-adult viability than *C. elegans* at 15°C, 20°C, and 25°C (Wilcoxon rank sum test p<0.001 in all comparisons; Fig. 5). No detectable differences in *C. inopinata* viability were found between 20°C, 25°C, and 30°C (median viability of 0.84, 0.79, and 0.88, respectively; Wilcoxon rank sum test W=50 p=0.060, W=70 p=0.62; Fig.5), but *C. inopinata* is less viable at 15°C (median viability of 0.63; Wilcoxon rank sum test p≤0.030 for all comparisons). As *C. inopinata* fecundity is also higher at warmer temperatures (Fig. 4a), these temperature-specific fitness patterns are consistent with its subtropical natural context of fresh Okinawan *Ficus septica* figs.

### Most *C. elegans* genes with life history phenotypes conventionally associated with large body size intersect with only one phenotype

**Fig. 6:**
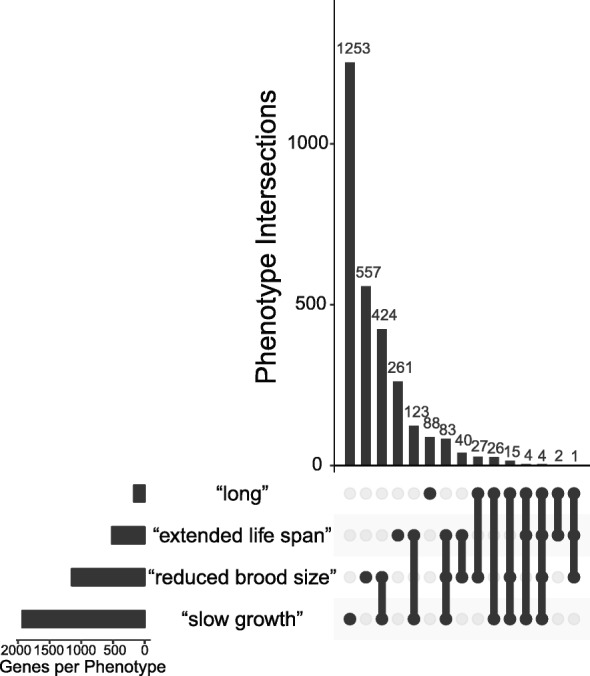
Intersection of relevant life history trait phenotypes in *C. elegans* protein-coding genes. In *C. elegans*, many genes that can increase body length, slow development, extend lifespan, or reduce fecundity when defective do not also promote correlated changes in life history traits often associated with increased body size. Matrix layout plot shows intersections of *C. elegans* genes among four WormBase phenotype terms [48] (“long,” “extended life span,” “reduced brood size,” “slow growth”; plot generated in R with the *UpSetR* package [86]). Most genes intersect with only one phenotype (the first, second, fourth, and sixth columns from the left), whereas only four genes display all four phenotypes. Most protein-coding genes in *C. elegans* do not have any reported phenotypes (also see analysis in [49]). See methods for more details.

Life history syndromes are often thought to be driven by trade-offs resulting from antagonistic pleiotropy [46]. How often are pleiotropic effects observed in life history traits in *C. elegans*? The *C. elegans* genomic database WormBase [47] has collected gene-specific information regarding the biological consequences of mutation and RNAi exposure as “phenotype” terms, which constitute a formal ontology used to describe phenotypes associated with genes [48]. To explore the extent of pleiotropy underlying life history syndromes in *C. elegans*, we measured the amount of overlap among four WormBase database phenotypes that resemble life history traits associated with large body size (“long,” “extended life span,” “reduced brood size,” and “slow growth”) in *C. elegans* protein-coding genes (Fig. 6). As previously shown [49], most *C. elegans* protein-coding genes do not have any reported phenotypes (42% or 8,585/20,209). 14% of *C. elegans* protein-coding genes (2,908/20,209) had at least one of the four life history phenotypes. Of these, the vast majority (74% or 2,159/2,908) intersected with only one of the four phenotypes (Fig. 6). This suggests that these traits are potentially largely genetically decoupled in this system and that pleiotropy need not underlie the evolution of life history strategies.

## Discussion

Possibly because it is both obvious and easy to measure, body size variation has been studied extensively for centuries. The range in body size across the tree of life is so immense as to demand explanation (21 orders of magnitude [16, 50]), and this incredible diversity has spawned a vast and rich literature attempting to comprehend its origins and maintenance. One major conclusion from this research program is that body size is correlated with nearly every trait, such that long-established relationships between body size and growth, reproduction, and lifespan underscore a prominent role for body size in the evolution of life histories [14, 15, 50]. Here, we found that an exceptionally large and ecologically divergent close relative of *C. elegans* exhibits slow growth and low fecundity across a range of temperatures yet is not long lived. Together with the extensive *C. elegans* literature and the foundations of life history theory, these observations can contribute to our understanding of the causes and consequences of large-scale changes in body size and ecological divergence.

### The impact of ecological divergence on life history syndromes

Changes in ecological context are expected to impact life history traits. Here, we described the life history traits of *C. inopinata*, an organism that occupies an exceptional ecological niche when compared with its close relatives. Most *Caenorhabditis* species, including *C. elegans*, thrive in rotting plant material [36]. *C. inopinata* proliferates in fresh *F. septica* figs, living in close association with its pollinating fig wasps [34, 37]. How might this dramatic shift in ecological context explain the patterns of life history traits observed here?

*C. inopinata* grows at nearly half the rate as *C. elegans* (Fig. 1). One possible explanation for the divergence in developmental rate could be its novel natural context. Most *Caenorhabditis* proliferate in the ephemeral environments of rotting vegetation [36]; it is thought that the rapid turnover and spatial patchiness of its bacterial food resources has driven its rapid development, high fecundity, and its resource-dependent decision to enter the dispersal dauer larval stage [51]. *C. inopinata*, conversely, grows in the presumably more stable environment of the fig lumen and obligately disperses on pollinating fig wasps [34, 37]. Figs generally take weeks to develop [52], although it is unclear how many generations of worms occur within a single fig. It is then possible that the extreme divergence in developmental rate might be connected to differences in the transience of resource availability between these environments. Furthermore, as *C. inopinata* animals disperse to new figs via pollinating wasps [37], their life cycle is necessarily closely tied to patterns of wasp development and emergence, consistent with correlations among *Ceratosolen* fig wasp and *C. inopinata* developmental stages that have been found in previous field studies [37]. This contrasts with *C. elegans* and its other close relatives, who disperse on a plethora of invertebrate carriers (including isopods, myriapods, and gastropods) [53]. Future longitudinal field studies of single fig trees at finer temporal resolution will be required to determine the relative paces of fig, fig wasp, and nematode development in nature and to test hypotheses regarding the ecological drivers of heterochrony.

Overall, *C. inopinata* reveals a lower viability (Fig. 5) and fecundity (Fig. 4) than *C. elegans* in laboratory conditions, although lifetime access to males greatly improves *C. inopinata* fecundity (Fig. 4b). How might its unique ecology underlie these patterns? A particularly interesting avenue to pursue is based on the observation that wild bacteria associated with *Caenorhabditis* can have both positive or negative influences on fecundity and growth [54, 55] and that different species of *Caenorhabditis* are associated with different microbes in nature [54]. Thus the nutritional environment can have a profound effect on fitness and life history traits. The natural microbial food of *C. inopinata* is currently unknown. As *C. inopinata* exhibits reduced gonads in laboratory culture [35], it may be experiencing nutritional deficiencies. The reduced fecundity of *C. inopinata* may then reflect a plastic response to an adverse environment as opposed to a trade-off with increased body size. The potential influence of natural microbial associates of *Ficus septica* figs on *C. inopinata* fitness affords an exciting opportunity for future research.

The large body size of *C. inopinata* could also be more directly connected to its novel environmental context. Body size is broadly correlated with fecundity across nematode phylogeny [56–58], and the fig microcosm may represent an environment with less selective pressure on body size than rotting vegetation. Hence *C. inopinata* may be large because its ecological context reveals relaxed selection on body size compared to its close relatives. Such pressures may entail predation or pathogens—various mites, fungi, bacteria, and viruses are known to prey on or infect *Caenorhabditis* [53]. Figs maintain many defenses against antagonistic invertebrates and microorganisms [59–61], and it is likely that this environment harbors a less diverse community than rotting vegetation. In this case any trade-offs among predation or infection avoidance and body size would be lifted, facilitating body size change resulting from selection on increased fecundity. However, within *C. elegans*, no evidence for correlations between body size and fecundity was found after artificial selection for large body size [25]. Thus it is entirely possible that such trade-offs are not driving body size change in this case and that other factors (adaptive or not) are responsible upon shifting to the fig environment. Further studies explicitly addressing the possible ecological determinants of body size change will be needed to delineate these possibilities.

### Developmental timing and body size

It makes intuitive sense that larger organisms should develop more slowly. Being more massive, presumably more cell divisions and/or biosynthetic reactions must take place for their construction and it therefore follows that their development should take longer than smaller organisms. And this intuition bears out across vast phylogenetic distances: from bacteria to sequoias, body size covaries with generation time [50]. Here, we found that in all temperatures, *C. inopinata* grows nearly half as quickly as *C. elegans*, consistent with previous observations (Fig. 1; [34, 35]). Indeed, *C. inopinata* needs to be grown at 30°C to approach a rate of development comparable to that of *C. elegans* when grown at 20°C. Thus, the observation that this very large species also develops much more slowly than its close relatives is in line with decades of allometric studies. Further, as cell size is coordinated with cell division decisions in multiple organisms [62, 63], body size change could occur even in the absence of cell number change through the modification of cell cycle timing. This may explain the case of *C. inopinata*, as previous observations observed no change in cell number despite its large size and slow development [35].

However, there are reasons to suspect slow development may not underlie large body size in this case. It has been argued that the allometric trends observed in birds and mammals cannot be easily extended to poikilotherms because of difficulties in comparing physiological time due to rapid change in metabolic rates [16]. More notable is the common observation that developmental timing can be decoupled from body size in *C. elegans*. Most mutations in *C. elegans* that extend body length do not also slow the rate of growth: only 29% of the genes in the *C. elegans* genome known to control body length also promote slower development (Fig. 6). Furthermore, experimental evolution and mutation accumulation studies in *C. elegans* and *C. briggsae* have not generally reported correlated changes in body size and developmental timing [25, 27, 28, 64]. Thus, it appears that body size and rate of growth need not be strongly coupled in *Caenorhabditis* and that the relationship between these traits observed in *C. inopinata* may not necessarily be causative. Furthermore, as mentioned above, the slow growth of *C. inopinata* may also be better understood with respect to its natural ecological context (or could also be due to nutritional deficiencies in a laboratory context); framing the causes of heterochronic change with respect to this context is an exciting avenue for future studies.

### Reproduction and body size

The relationship between body size and reproduction varies both within and between taxa. In birds and mammals, larger species tend to have lower fecundities than smaller species [15]. Conversely, body size appears to be positively correlated with fecundity in insects [65] and nematodes [56–58]. *C. inopinata* was generally found to have lower brood sizes than *C. elegans* across a range of temperatures (Fig. 4a), although continuous mating greatly improves fecundity in *C. inopinata* (Fig. 4b). The relatively low fecundity of *C. inopinata* is then incongruent with patterns of fecundity and body size that have been previously reported among nematodes [56–58]. *C. inopinata*’s gonochoristic mode of development cannot explain its low brood size, as multiple male/female species of *Caenorhabditis* have been reported to have higher brood sizes [42, 43, 66–69]. However, the sperm-limited fecundity of *C. inopinata* (Fig. 4b) is consistent with previous observations with the gonochoristic *C. remanei* [42, 43]. It is possible that the evolution of extreme body size in the case of *C. inopinata* reveals a trade-off with reproductive output, wherein resources usually allocated to progeny have instead been shifted to increase self-maintenance and growth. Yet most genes known to regulate body length in the *C. elegans* genome do not appear to have a pleiotropic role in brood size (only 28% do; Fig. 6). This is also consistent with experimental evolution studies in *Caenorhabditis* [25], wherein fecundity and body size do not necessarily trade-off. So again, the precise causal relationship here bears further study. Additionally, as mentioned above, patterns of fecundity in *C. inopinata* may also be better understood with respect to its natural environment. It is possible that *C. inopinata* may be experiencing nutritional deficiencies in the laboratory context, and future studies with natural associated microbes will help to inform this possibility.

### Lifespan and body size

Lifespan is often positively correlated with body size, and from an allometric perspective is usually thought to be regulated by variation in developmental and metabolic rates [15, 17]. And although the age of maturity is sensitive to selection under a range of trait distributions in life history theory [1], from an evolutionary perspective it is thought that late-life traits are generally not subject to selection as its strength falls to zero once reproduction ends [3]. Despite its large size and slow development, *C. inopinata* was found to have only a marginally longer lifespan than *C. elegans* (Fig. 2). And, when differences in developmental timing and reproductive mode are taken into account, *C. inopinata* adult lifespan is not significantly different from that of *C. elegans* (Fig. 2b). The lack of lifespan change in this system is consistent with the view that lifespan is under weak selection, as *C. inopinata* has experienced dramatic change in many other traits under its novel ecological context [34, 35, 37]. Indeed, most lifespan-extending mutations identified in *C. elegans* have not been associated with pleiotropic effects on body size (Fig. 6). Similarly, experimental evolution studies in *C. elegans* show no correlated responses in lifespan upon artificial selection on early fecundity [32] and body size [25]. Additionally, no relationships between lifespan and fecundity have been found in mutation-accumulation lines [24] or among wild isolates [26]. These observations are inconsistent with the antagonistic pleiotropy explanation of aging, which posits that the greater fitness contribution of early life survival and reproduction leads to late life deterioration because of negative genetic correlations of these traits [70]. Rather, lifespan appears to be possibly largely uncoupled from fitness-related traits in this group, consistent with the unchanged longevity observed in *C. inopinata*. However, the nutritional caveats in this system noted in the above interpretation of observed patterns of fecundity also apply here. It is possible that *C. inopinata* will be longer-lived under different rearing conditions, and measurements of lifespan of *C. inopinata* raised on bacterial food originating from its natural context need to be performed.

### Variation, inbreeding, and fitness-related traits

The observations reported here constitute comparisons of two species with each represented by one genetic strain. How does this fact impact the implications of this work discussed here? From a comparative phylogenetics perspective, this sample size is simply insufficient to make broad generalizations about patterns of life history trait covariation in the *Caenorhabditis* genus. It remains possible that *C. inopinata* represents an outlier that defies meaningful biological trends that we would otherwise not capture because of our limited phylogenetic sample. Furthermore, as we have only interrogated one strain of *C. inopinata*, this strain may also not be representative of this species as such. Ultimately, broad sampling and measures across *Caenorhabditis* phylogeny will be needed to make solid claims along these lines. However, can previous observations of variation in fitness-related traits in *Caenorhabditis* help to overcome this limitation or better inform these results?

*C. inopinata* is an exceptional *Caenorhabditis* species with respect to its large body size [34, 35], and most members of the *Elegans* group are difficult to distinguish morphologically [71, 72]. But, as mentioned above, there is variation both within and between *Caenorhabditis* species in fecundity (Additional file 1: Figures S5-S6; Additional file 2) and developmental rate (Additional file 1: Figures S7-S8; Additional file 3). Among four recent studies that measured fecundity in *Caenorhabditis* (including this study) [38, 42, 73] , which includes 24 strains among four species, our estimate of *C. inopinata* fecundity at 20°C is the lowest (Additional file 1: Figures S5-S6). But as *C. inopinata* and other gonochoristic *Caenorhabditis* are sperm-limited (Fig. 4) [42, 43], this is likely an underestimate of its reproductive capacity. However, even limited access to males can maintain brood sizes in *C. remanei* wild isolates that exceed those observed in many strains of selfing species (Additional file 1: Figures S5-S6) [42], including *C. elegans* N2, which is considered a domesticated laboratory strain [74]. With respect to previous studies of developmental timing, *C. inopinata* is even more extreme in its divergence from its close relatives, developing at about half the rate as the next slowest strain (*C. tropicalis* JU1630) among those considered in two previous publications (Additional file 1: Figures S7-S8) [38, 75]. Thus a broader phylogenetic context with more *Caenorhabditis* species and strains also suggests that this *C. inopinata* strain harbors an exceptionally slow developmental rate and a low (but sperm-limited) fecundity in addition to its large body size.

Gonochoristic *Caenorhabditis* species are susceptible to inbreeding depression [76, 77]. Could *C. inopinata* strain NKZ2 have experienced inbreeding depression during laboratory culture that impacts estimates of fitness-related traits? This possibility cannot be definitively ruled out, and an important caveat of these results is that inbreeding depression may influence the patterns of life history traits observed here. However, there are reasons to suspect inbreeding depression may not be a major influencing factor in this case. Primarily, *C. inopinata* NKZ2 is a wild isolate that has not been deliberately inbred, and it is a strain derived from multiple founding individuals [35]. If inbreeding depression were the only cause of declines in fitness-related traits, then *C. inopinata* NKZ2 should be expected to have higher fecundity than isofemale wild isolates and inbred lines of other gonochoristic species as it is derived from more than one founding individual. As this is not the case (Additional file 1: Figures S5-S6), this suggests that inbreeding depression alone may not be driving these patterns. Furthermore, the ecology of *C. inopinata* may render it less susceptible to inbreeding depression through its obligate dispersal on fig wasps [37]. Despite this, the embryo-to-adult viability of *C. inopinata* is low across multiple temperatures (Fig. 5), which is itself suggestive of inbreeding depression. As we did not measure larval survival directly, it is unclear at what stage animals are undergoing developmental arrest. Additionally, it is possible that behavior could also be driving these results—larvae that crawl off plates and do not mature will artificially deflate viability measurements. However, as dead embryos have been anecdotally observed in these cultures, embryonic lethality is likely driving at least some of these patterns. It also remains possible that the culture conditions designed for *C. elegans* are insufficient for the reliable development of this species, and native fig, wasp, or microbial factors may be needed for robust *C. inopinata* viability. Regardless, future studies that rear this species in more ecologically-relevant culture conditions, use *C. inopinata* inbred lines and wild isolates, and implement population genomic approaches in natural populations will be needed to disentangle these possibilities.

### Pleiotropy and life history syndromes

Here we note relationships among life history traits across two species of *Caenorhabditis*. What role does pleiotropy play in the patterns observed here, and are life history syndromes the result of indirect selection, direct selection on multiple characters, or both? Because this study does not directly interrogate the genetic basis of these traits, the underlying genetic causes of these relationships are undetermined. However, the vast background information associated with the *C. elegans* model system can provide context to generate hypotheses regarding the evolution of life history strategies. How many genes have pleiotropic effects on multiple life history traits in *C. elegans*? As discussed above, most genes associated with one of four life history WormBase phenotypes [48] that might be associated with large body size (“slow growth”, “reduced brood size,” “extended life span,” and “long”) do not intersect with one another (Fig. 6). That is, most genes with any of these phenotypes are associated with only one of the four (74%; Fig. 6), and only a fraction of them reveal evidence of pleiotropic effects (26%; Fig. 6). Thus these life history traits appear to be largely genetically decoupled in this group, and pleiotropy need not underlie the correlated evolution of these traits. However, as some genes do influence multiple phenotypes (Fig. 6), pleiotropy may still contribute to the evolution of life history syndromes in this case. Furthermore, as many *C. elegans* studies are largely concerned with only a few phenotypic traits of interest, these results are likely to underestimate the extent of pleiotropy among *C. elegans* genes. Regardless, future work investigating the genetic bases of these traits in *C. inopinata* will be needed to understand the role of pleiotropy in shaping life history syndromes.

### Temperature-dependent patterns of fitness-related traits in C. Inopinata

Notably, *C. inopinata* was more fit at higher than lower temperatures (Fig.4a, Fig. 5). Temperature-dependent plasticity of fitness-related traits varies both within and between species in *Caenorhabditis*, and these patterns often coincide with ecological context. Within *C. briggsae*, there are definable clades that are genetically structured by latitude [78, 79], and these wild isolates reveal temperature-dependent patterns of fecundity that are consistent with their geographical origin [80]. Additionally, the tropical species *C. nigoni* [66, 81] and *C. tropicalis* [82] have higher fitness at warmer temperatures. As *C. inopinata* has only been found in the subtropical islands of Okinawa [34, 35], its temperature-dependent patterns of fitness are consistent with these previous observations. And further, the temperatures where *C. inopinata* has shown the highest fitness here are comparable to natural *Ficus septica* fig temperatures measured in nature [37]. Additionally, *C. inopinata* needs to be grown at 30°C to approach a rate of development comparable to that of *C. elegans* when grown at 20°C. Could the slow growth of *C. inopinata* more appropriately be interpreted as an adjustment of optimal developmental timing imposed by its subtropical environment? This explanation is appealing as thermal plasticity in growth is widespread in ectotherms, and wild *F. septica* fig interiors were found to harbor temperatures of 29°C on average [37]. However, if temperature were a major driver of a universal, optimal developmental rate in *Caenorhabditis*, then we would expect to see much slower development in tropical strains and species than has been reported (Additional file 1: Figure S8). Indeed, there are no detectable developmental timing differences between tropical and temperate strains of *C. briggsae* (Additional file 1: Figure S8) [38], which nonetheless do reveal clade-specific, temperature-dependent differences in fecundity [80]. And although *C. tropicalis*, which is typically found in warmer climates than *C. inopinata* [51, 83], harbors a slower developmental rate than *C. elegans* (Additional file 1: Figure S7-S8) [38], it remains far faster than that of *C. inopinata* (Additional file 1: Figure S7-S8). Thus, it seems more likely that the slow growth of *C. inopinata* is connected to its novel ecological context in *F. septica* figs, its exceptionally large body size, or both, rather than its subtropical locality alone. Regardless, as a close relative of *C. elegans*, this species is well positioned for uncovering the genomic bases of temperature adaptation.

## Conclusions

Body size and ecological divergence are major drivers of evolutionary change in multiple taxa, and such changes often co-occur with widespread change in life history traits. Here, we examined the life history traits of a large, ecologically-divergent close relative of *C. elegans*. We found that *C. inopinata* develops at nearly half the rate as *C. elegans*, revealing a likely trade-off between growth and body size. Conversely, longevity does not evolve as part of correlated response to selection on body size in this system, consistent with previous studies and indicative of genetic decoupling of longevity from other life-history traits. Future studies that situate these systems within their natural ecological contexts will be needed to fully disentangle matters of cause and effect among the traits that constitute life history strategies. Taken together, these observations reveal that drastic change in ecological context and body size do not necessarily have an all-encompassing impact on life history syndromes.

## Methods

### Strains and maintenance

Animals were maintained on Nematode Growth Media (with 3.2% agar to discourage burrowing) supplemented with *Escherichia coli* strain OP50-1 for food. The *C. inopinata* wild isolate strain NKZ2 [35] was utilized for all observations in this report. *C. elegans* N2 and the obligate outcrossing *C. elegans fog-2(q71)* JK574 [41] mutant strain were also used for most comparisons. Notably, *C. elegans* is hermaphroditic, while *C. inopinata* is male/female or gonochoristic. This makes interspecific comparisons problematic. Thus the *fog-2(q71)* mutation, which prevents spermatogenesis only in hermaphrodites but promotes no obvious somatic defects in either sex [41], was used to control for differences in reproductive mode in various comparisons of life history traits.

### Developmental timing

The timing of four developmental milestones (hatching, fourth larval stage (L4), adult stage/young adulthood, and the onset of reproduction/reproductive adulthood) was measured at four temperatures: 15°C, 20°C, 25°C, and 30°C. For synchronization, mid-stage embryos (blastula to 1.5 fold stage) were picked from plates cultured at 25°C to new plates and then shifted to the given rearing temperature. Plates were then monitored hourly (for hatching) and then daily (for L4, young adulthood, and reproductive adulthood) for the onset of developmental milestones. Male tail and female/hermaphrodite vulva morphologies were used to define L4 and young adult stages. The onset of reproduction was scored only among females and hermaphrodites by the presence of embryos in the uterus. Plates were assayed until the number of individuals at or older than a given milestone did not increase for two hours or days. Animals who failed to reach a given milestone were not used for subsequent analysis. For analysis, animals were plotted by their developmental status (“0” = yet to reach milestone; “1” = reached milestone) over time and logistic regression was used to estimate the median time to a given event via the “glm” function (using a binomial distribution) in the R statistical language. This models approach was used for hypothesis testing and for calculating 95% confidence intervals (see Additional file 4; data are available in Additional files 5 and 6).

### Lifespan

Synchronized animals were generated by allowing gravid females/hermaphrodites (20 *C. elegans* hermaphrodites or *C. elegans fog-2(q71)* pseudo-females per plate; about 100 *C. inopinata* females per plate) to lay for 2-3 hours. After a few days, synchronized L4 virgin females/hermaphrodites were moved to new plates, with about 30 nematodes per plate. All animals were transferred every day for the first 4-5 days of adulthood as hermaphrodites reproduced. Subsequently, animals were scored every 1-3 days as either living or dead up until the point that all animals had died. All measurements were performed at 25°C. The number of days alive after egg-laying was taken as the measure of total lifespan. Lifespan and longevity studies in *C. elegans* are often concerned with the basis of aging, which is generally thought to largely occur in adulthood after developmental growth [84]. Thus we report here both total lifespan (starting at embryogenesis) and adult lifespan (starting at the onset of maturation). As *C. inopinata* and *C. elegans* display different rates of developmental growth, this also allows a comparison of the rate of aging in adults that accounts for this difference. Adult lifespan was taken as the total lifespan minus two (*C. elegans*) or four (*C. inopinata*) days, as *C. inopinata* develops at about half the rate as *C. elegans*. Statistical analyses were performed as in [38], with the *survival* package for the R statistical language being used to generate survivorship curves and the *coxme* package being used to generate Cox proportional hazard models and perform hypothesis tests (see Additional file 4; data are available in Additional file 7).

### Fecundity

Daily offspring production was measured following overnight mating and under continuous exposure to males. For all observations, L4 *C. inopinata* NKZ2 and *C. elegans fog-2(q71)* animals raised at 25°C were isolated and raised for one (*C. elegans*) or two (*C. inopinata*) days to adulthood (see above). For overnight mating, single adult females/pseudo-females were shifted to the given experimental rearing temperature and mated with six males overnight. Brood sizes were measured at 15°C, 20°C, 25°C, and 30°C. The next day males were removed. Every day, embryos and larvae were counted, and egg-laying females were moved to new plates. New progeny were scored until females stopped laying for at least one (*C. elegans*) or two (*C. inopinata*) consecutive days. Continuous mating conditions were similar, except that single females were always in the presence of six males. Males that crawled up the side of the plate or otherwise died before the female stopped laying embryos were replaced with young adult males. The continuous mating observations were performed at 25°C. The instantaneous rate of natural increase [1] was calculated in Python as in [85] using life tables for *C. elegans* and *C. inopinata* constructed from the viability, fecundity, and lifespan data developed here (see Additional file 8; data are available in Additional files 9, 10, 11 and 12).

### Embryo-to-adult viability

Nematode embryos were synchronized by allowing gravid females/hermaphrodites (20 *C. elegans* hermaphrodites or *C. elegans fog-2(q71)* pseudo-females per plate; about 100 *C. inopinata* females per plate) to lay for 2-3 hours. After the parents were removed, the number of embryos per plate were counted, and the plates were shifted to their respective rearing temperatures (15°C, 20°C, 25°C, or 30°C). L4 and adult worms were counted 4-5 days later. This fraction of mature worms/initial worm embryos was reported as the viability. Data are available in Additional file 13.

### Intersection of WormBase phenotypes related to life history traits among *C. elegans* protein-coding genes

Functional annotations for all *C. elegans* protein-coding genes were retrieved using the simplemine tool in WormBase (http://www.wormbase.org/tools/mine/simplemine.cgi, link labeled “query all *C. elegans*”; Additional file 14). Genes with mutant or RNAi phenotypes “long,” “slow growth,” “extended life span,” and “reduced brood size” were extracted, and a spreadsheet denoting the intersection of these four phenotypes for every gene that included at least one of these phenotypes was created with Linux (see Additional file 15 for software) and Perl (https://github.com/religa/stats/blob/master/merge) tools (see Additional files 14 and 15 for data). The *UpSetR* package [86] was used to make Fig. 6 with this data (Additional file 4).

## Additional files

Additional file 1: **Figure S1.** Total lifespan models with 95% confidence intervals. **Figure S2.** Adult lifespan models with 95% confidence intervals. **Figure S3.** Patterns of failed crosses across mating conditions and temperatures. **Figure S4.** *C. inopinata* has lower brood sizes than *C. elegans (fog-2)* in continuous mating conditions after removing failed crosses. **Figure S5.** Comparison of intra- and interspecific variation in *Caenorhabditis* fecundity in four recent studies (with data colored by publication). **Figure S6.** Comparison of intra- and interspecific variation in *Caenorhabditis* fecundity in four recent studies (with data colored by strain locality). **Figure S7.** Comparison of intra- and interspecific variation in *Caenorhabditis* age of maturation in three studies (with data colored by publication). **Figure S8.** Comparison of intra- and interspecific variation in *Caenorhabditis* age of maturation in three studies (with data colored by strain locality). **Table S1.** Estimates of median time of developmental events. (PDF 682 kb)

Additional file 2: Fecundity_metadata.tsv. Fecundity metadata collected for Additional file 1: Figure S5-S6. (TSV 42 kb)

Additional file 3: Developmental_timing_metadata.tsv. Developmental timing metadata collected for Additional file 1: Figure S7-S8. (TSV 6 kb)

Additional file 4: Models_hypothesis_tests.R. Software for generating models and statistics. (R 52 kb)

Additional file 5: Hatch_time_data.tsv. Developmental timing data for hatching. (TSV 7 kb)

Additional file 6: Postembryonic_milestone_time_data.tsv. Developmental timing data for postembryonic milestones. (TSV 37 kb)

Additional file 7: Lifespan_data.tsv. Lifespan data. (TSV 42 kb)

Additional file 8: Estimate_r.py. Software for estimating the rate of population increase. (PY 14 kb)

Additional file 9: Reproductive_duration_data.tsv. Reproductive duration data. (TSV 15 kb)

Additional file 10: Fecundity_lifetime_access_data.tsv. Fecundity with lifetime access to males data. (TSV 2 kb)

Additional file 11: Fecundity_overnight_mating_data.tsv. Fecundity with one overnight mating data. (TSV 2 kb)

Additional file 12: Life_tables.tsv. Data used for estimating the rate of population increase. (TSV 14 kb)

Additional file 13: Viability_data.tsv. Viability data. (TSV 1 kb)

Additional file 14: Wormbse_simplemine.txt. Data retrieved from WormBase for generating upset_plot_input.tsv (Additional file 16). (TXT 5290 kb)

Additional file 15: Wormbase_phenotype_intersections.sh. Software for generating phenotype intersection UpSet plot data from a WormBase Simplemine tab-delimited file. (SH 6 kb)

Additional file 16: Upset_plot_input.tsv. Data for generating an UpSet plot as in Figure. (TSV 453 kb)

## Abbreviations

CI: Confidence interval
Fog: Feminization of germline
GLM: Generalized linear model
L4: Fourth larval stage
LRT: Likelihood ratio test
