# Supplementary material for: A large close relative of C. elegans is slow-developing but not long-lived
Source: BMC Evol Biol. 2019 Mar 11;19:74. doi: 10.1186/s12862-019-1388-1 (PMC6416856; doi:10.1186/s12862-019-1388-1)
Supplement: Supplementary file 1 — Figure S1. Total lifespan models with 95% confidence intervals. Figure S2. Adult lifespan models with 95% confidence intervals. Figure S3. Patterns of failed crosses across mating conditions and temperatures. Figure S4. C. inopinata has lower brood sizes than C. elegans (fog-2) in continuous mating conditions after removing failed crosses. Figure S5. Comparison of intra- and interspecific variation in Caenorhabditis fecundity in four recent studies (with data colored by publication). Figure S6. Comparison of intra- and interspecific variation in Caenorhabditis fecundity in four recent studies (with data colored by strain locality). Figure S7. Comparison of intra- and interspecific variation in Caenorhabditis age of maturation in three studies (with data colored by publication). Figure S8. Comparison of intra- and interspecific variation in Caenorhabditis age of maturation in three studies (with data colored by strain locality). Table S1. Estimates of median time of developmental events. (PDF 682 kb) [file 12862_2019_1388_MOESM1_ESM.pdf]

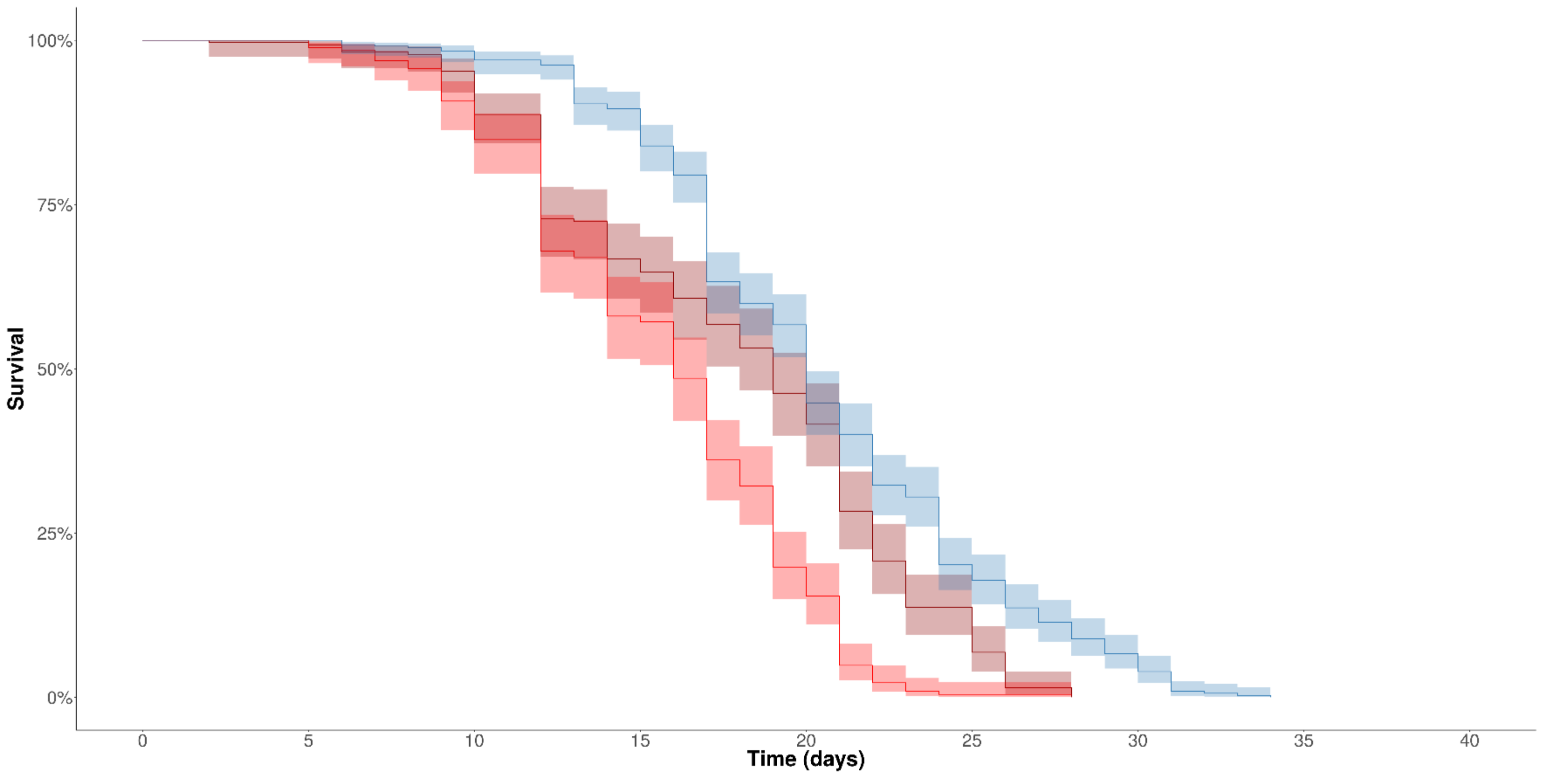

Figure S1. Total lifespan models with 95% confidence intervals. Bright red, *C. elegans* N2. Maroon, *C. elegans (fog-2)*. Blue, *C. inopinata*

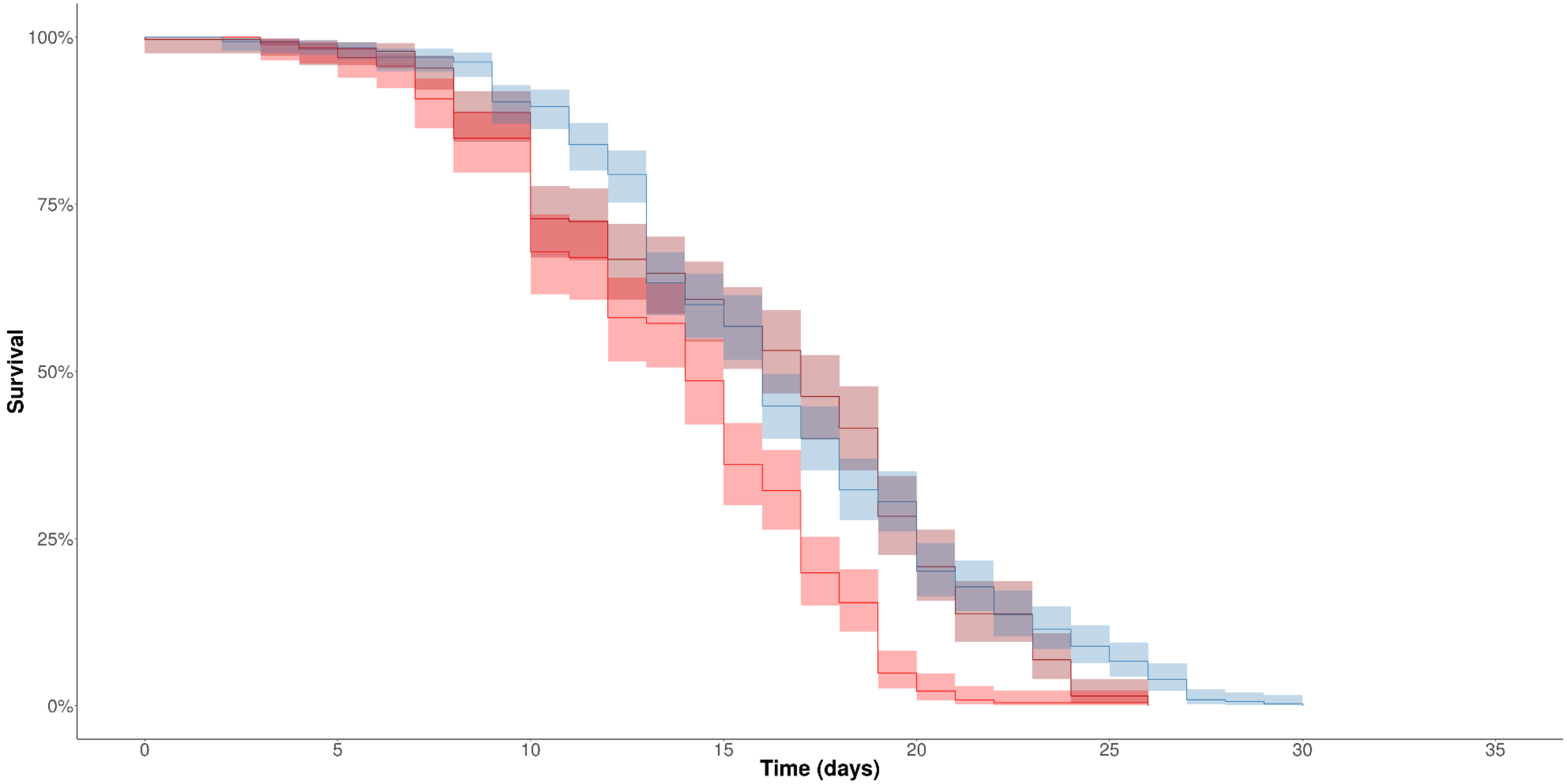

Figure S2. Adult lifespan models with 95% confidence intervals. Bright red, *C. elegans* N2. Maroon, *C. elegans (fog-2)*. Blue, *C. inopinata*

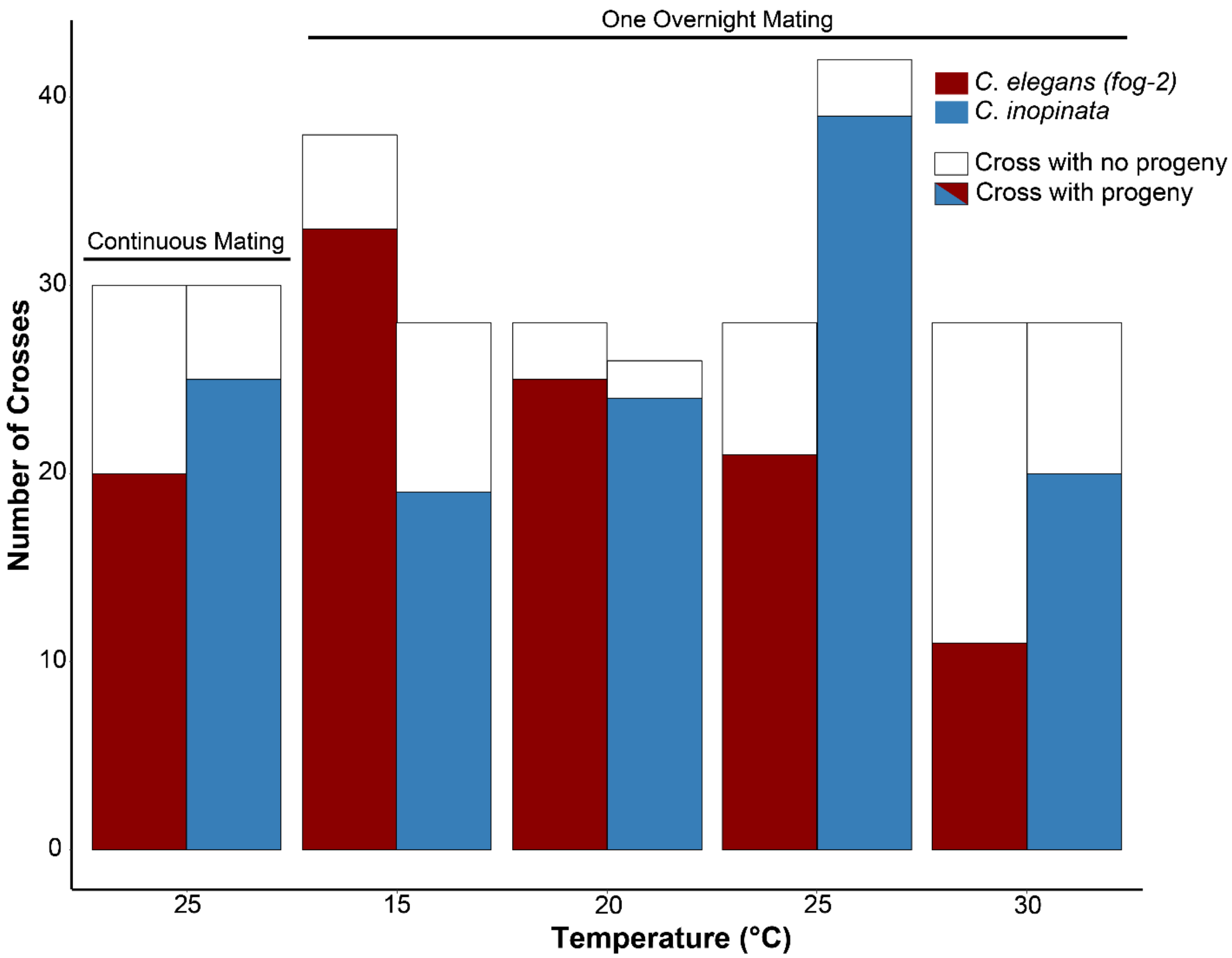

Figure S3. Patterns of failed crosses across mating conditions and temperatures. Only the overnight mating at 30°C showed a significant difference in failed cross fraction between species (Fisher's exact test $p=0.03$; all other crosses $p > 0.05$).

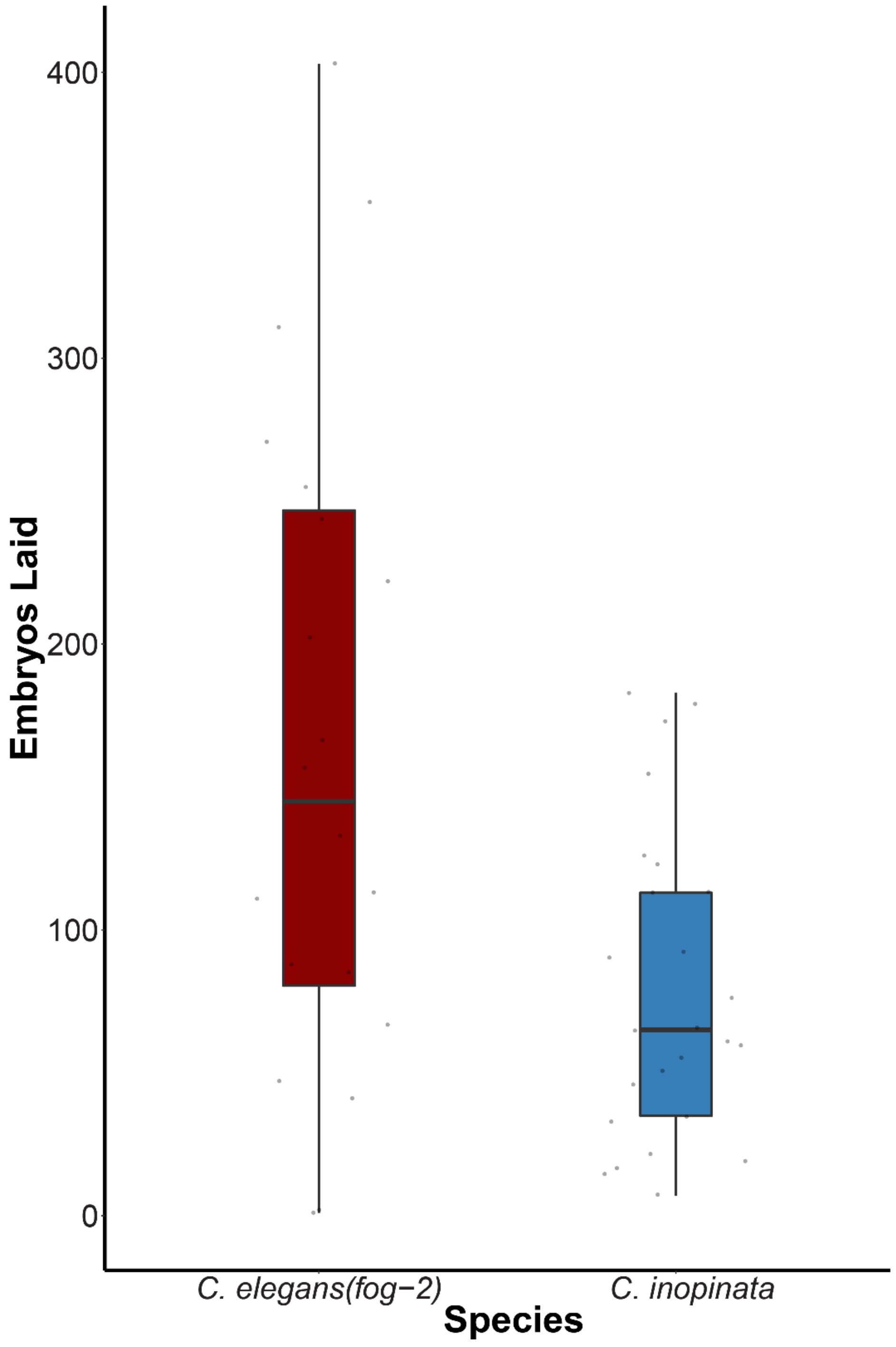

Figure S4. *C. inopinata* has lower brood sizes than *C. elegans (fog-2)* in continuous mating conditions after removing failed crosses. *C. elegans (fog-2)* N crosses=20; *C. inopinata* N crosses=25.

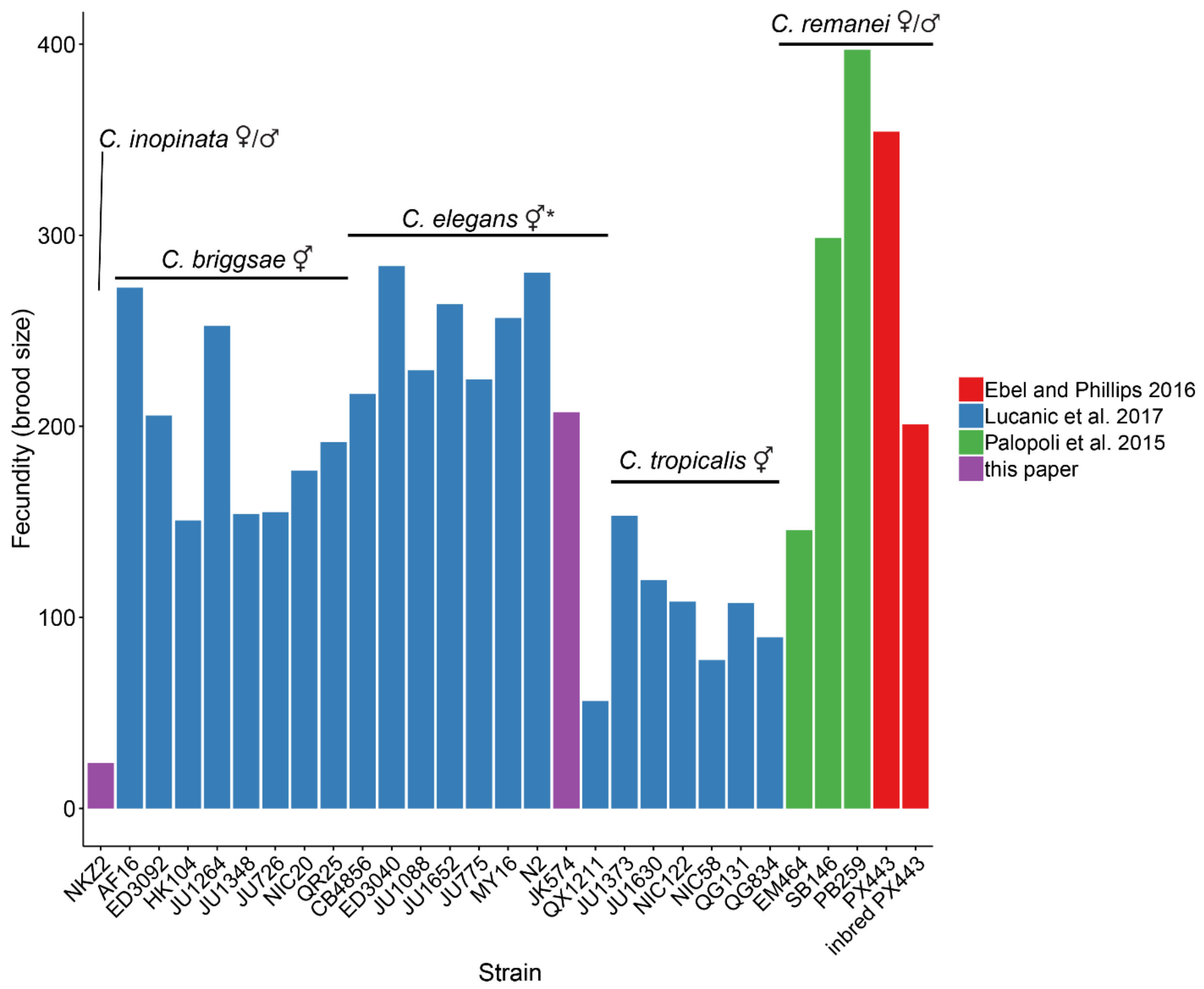

Figure S5. Comparison of intra- and interspecific variation in *Caenorhabditis* fecundity in four recent studies (with data colored by publication). ♀/♂, male/female species. ⚥, hermaphroditic species. **C. elegans* is a hermaphroditic species, but strain JK574 carries a mutation in *fog-2* which inhibits hermaphrodite spermatogenesis and is an obligate male/female line. These point estimates of brood size were retrieved from data reported in this paper or Dryad depositories. All observations were reported to be taken at 20°C. For estimates from Lucanic et al. 2017, only self-brood sizes were reported for hermaphrodites in the absence of males. These estimates are grand means of multiple fecundity measurements across three laboratories; N worm sample sizes range from 53-233 with an average of 79 per strain. Aside from *C. elegans* N2, which is a domesticated laboratory line, all strains are wild isolates. For Palopoli et al. 2015, only data for within strain crosses with 24 hours of male access were retrieved and means were plotted. Here, single females were mated with five males for 24 hours. All strains are wild isolates. N females EM464=14, SB146=19, PB259=14. For Ebel and Phillips 2016, data were retrieved to illustrate the impact of inbreeding in outcrossing *Caenorhabditis* species and means were plotted. For strain PX443, a deliberately outbred strain with high polymorphism originating from 26 wild isolates (Sikkink et al. 2014), brood sizes were determined for single male and single female crosses. For "inbred PX443" individual males or females derived from five generations of paired sibling inbreeding of PX443 were crossed with a non-inbred mate. N crosses PX443=291, inbred PX443=191. For this paper, only the overnight mating crosses with one female and six males at 20°C were included. For details regarding data see Additional Files 2 and 15, Lucanic et al. 2017, Palopali et al. 2015, and Ebel and Phillips 2016.

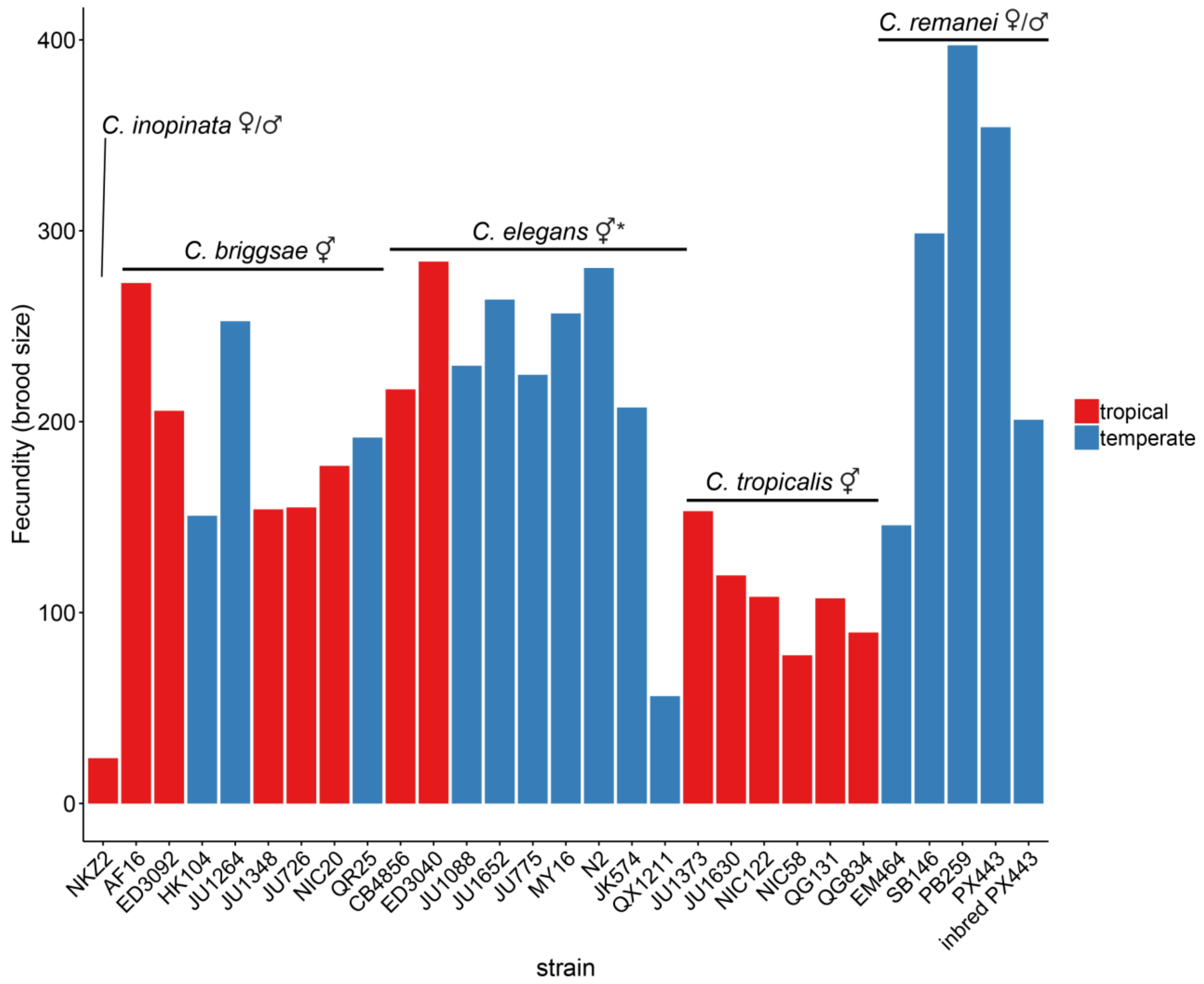

Figure S6. Comparison of intra- and interspecific variation in *Caenorhabditis* fecundity in four recent studies (with data colored by strain locality). Same data as Figure S5 colored by stain locality. “Tropical” here also includes subtropical localities, and “temperate” is any locality that is not tropical/subtropical. See legend of Figure S5 for discussion of the data included here.

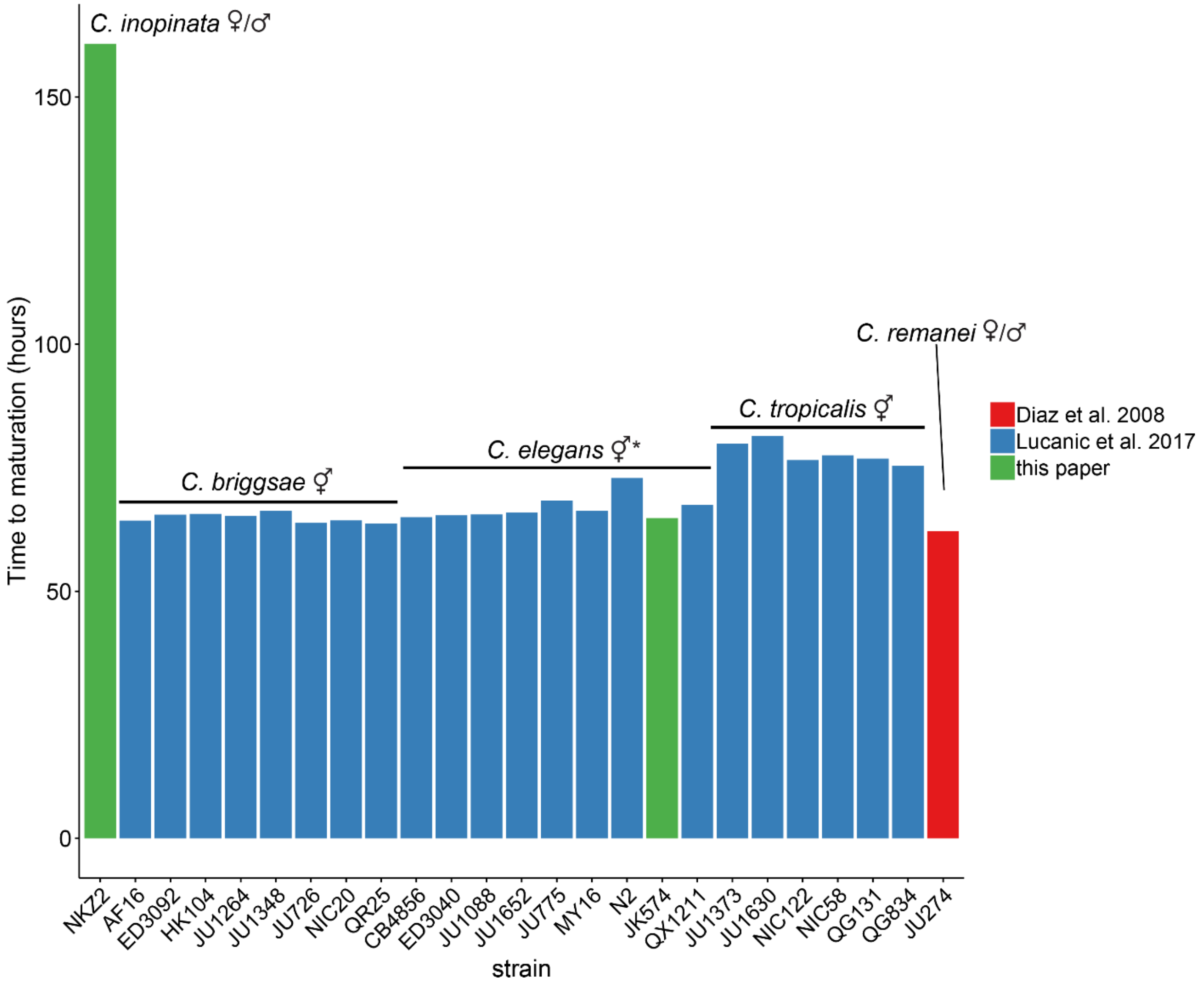

Figure S7. Comparison of intra- and interspecific variation in *Caenorhabditis* age of maturation in three studies (with data colored by publication). ♀/♂, male/female species. ⚥, hermaphroditic species. *C. elegans is a hermaphroditic species, but strain JK574 carries a mutation in fog-2 which inhibits hermaphrodite spermatogenesis and is an obligate male/female line. All observations were reported to be taken at 20°C. For estimates from Lucanic et al. 2017, data were retrieved from the Dyad depository and grand means of multiple developmental timing measurements across three laboratories were plotted. N worm sample sizes range from 20-258 with an average of 82 per strain. Aside from *C. elegans* N2, which is a domesticated laboratory line, all strains are wild isolates. For Diaz et al. 2008, the point estimate for wild isolate JU274 was taken from the first paragraph of the results ("Adult females and males exhibited similar dynamics; highest numbers were recorded at 2.59 ± 0.60 SD d"). For this paper, the median onset of reproduction at 20°C was used (see Table S1). Data are available in Additional File 16.

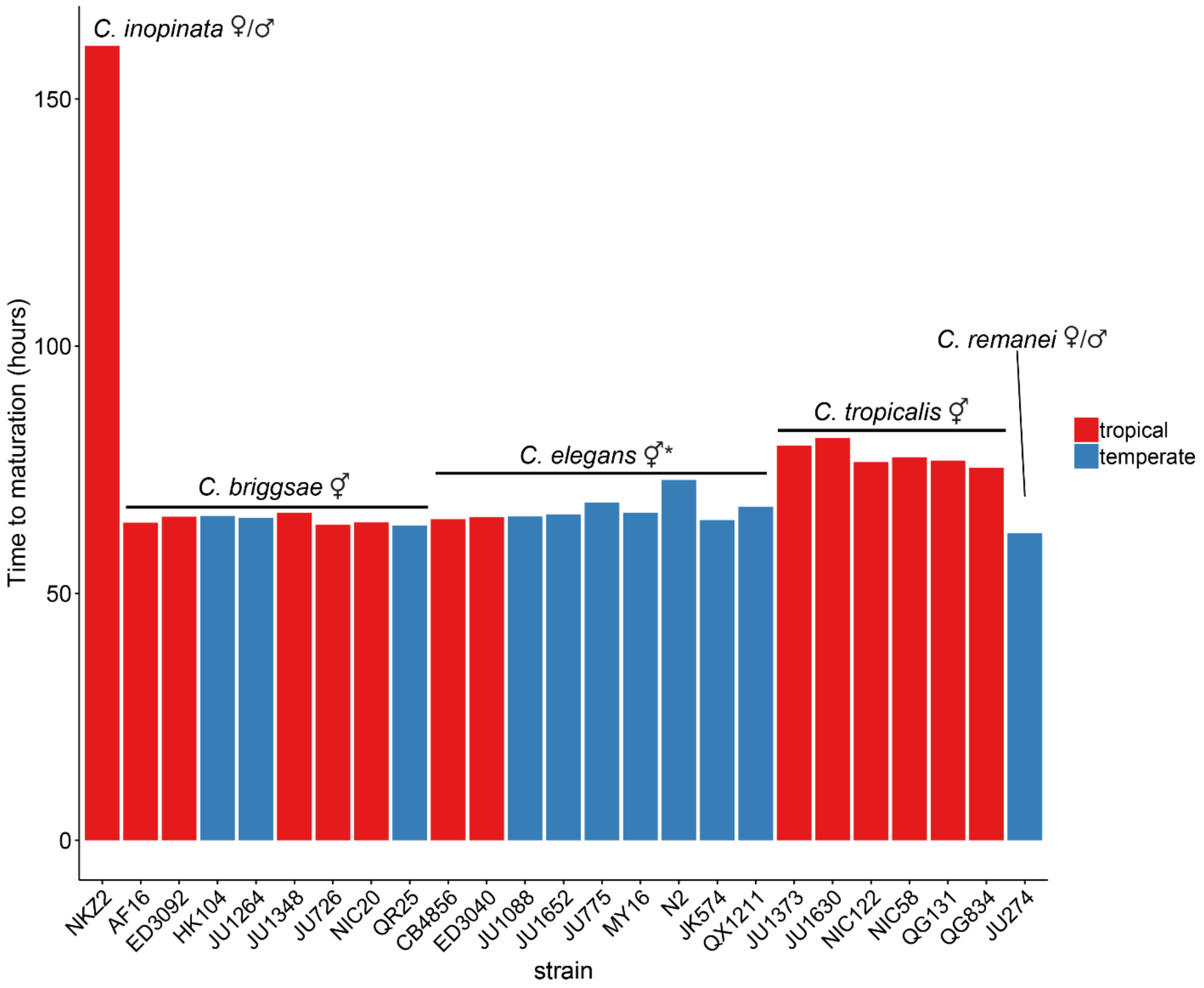

Figure S8. Comparison of intra- and interspecific variation in *Caenorhabditis* age of maturation in three studies (with data colored by strain locality). Same data as Figure S7 colored by stain locality. “Tropical” here also includes subtropical localities, and “temperate” is any locality that is not tropical/subtropical. Within *C. briggsae*, there is no significant difference in maturation time among tropical and temperate strains (Wilcoxon rank sum test W=8, p=1). See legend of Figure S7 for discussion of the data included here.

Temperature	15°C		20°C		25°C		30°C	
**Species**	*C. elegans*	*C. inopinata*	*C. elegans*	*C. inopinata*	*C. elegans*	*C. inopinata*	*C. elegans*	*C. inopinata*
**Hatching (hours)**	21.1<br>(18.1-24.7)<br>[2-50]	--	9.92<br>(8.05-12.2)<br>[2-26]	16.1<br>(13.9-18.7)<br>[2-28]	8.26<br>(7.82-8.36)<br>[2-26]	12.6<br>(10.2-15.6)<br>[6-26]	--	11.1<br>(9.64-12.9)<br>[2-32]
**L4 (days)**	3.7<br>(2.9-4.7)<br>[3-6]	--	2.1<br>(0.94-4.7)*<br>[2-4]	4.0<br>(3.1-5.0)<br>[3-9]	1.9<br>(0.80-4.7)*<br>[2-3]	2.8<br>(2.1-3.7)<br>[2-6]	--	2.2<br>(0.99-4.7)*<br>[2-5]
**Adulthood (days)**	4.9<br>(3.8-6.3)<br>[4-6]	--	2.6<br>(1.4-5.0)*<br>[3-4]	5.2<br>(4.0-6.7)<br>[5-9]	2.1<br>(0.92-4.7)*<br>[2-3]	3.5<br>(2.7-4.5)<br>[3-6]	--	2.5<br>(1.6-4.0)*<br>[2-5]
**Reproduction (days)**	5.4<br>(4.7-6.1)<br>[5-8]	--	2.7<br>(1.5-5.0)*<br>[3-4]	6.7<br>(5.7-7.8)<br>[5-9]	2.4<br>(2.0-3.0)<br>[2-4]	4.3<br>(3.8-5.0)<br>[3-6]	--	3.0<br>(1.4-6.5)*<br>[3-4]

Table S1. Estimates of median time of developmental events. Numbers represent the time at the inflection points of the logistic models of developmental timing shown in Figure 1. 95% confidence intervals of the median time to milestone in parentheses. Range of time to milestone in brackets. N2-derived strains of *C. elegans* are inviable at 30°C (Figure 5), and *C. inopinata* exhibits low fitness at 15°C (Figure 4A and Figure 5). *C. elegans (fog-2)* used for the onset of reproduction milestone; *C. elegans* N2 used for all other milestones. *Firth's biased-reduced logistic regression used to penalize instances of separation in developmental timing data (i.e., worms reached the milestone at the same time).
